# Localizing intramyocardially embedded left anterior descending artery during coronary bypass surgery: literature review

**DOI:** 10.1186/1749-8090-8-202

**Published:** 2013-10-30

**Authors:** Edem Ziadinov, Hilal Al-Sabti

**Affiliations:** 1 Department of Surgery, Cardiothoracic Surgery Division, Sultan Qaboos University Hospital, Al Khoud, Muscat, Sultanate of Oman

**Keywords:** Coronary artery bypass grafting, Embedded coronary artery

## Abstract

Proper detection of the deeply embedded left anterior descending artery remains a challenge. Many authors proposed different methods for artery identification, such as ultrasound Doppler, cineangiography, retrograde dissection overlying tissues, and exposure over the probe. Choice of the technique often depends on the surgeon's acquaintance and experience. The article compares and summarizes different procedures for the detection of intramyocardially located left anterior descending artery.

## Review

### Introduction

The left anterior descending (LAD) coronary artery is the most important artery for coronary revascularization procedures. It is predominantly grafted by left internal mammary artery, what is considered as a “gold standard” of coronary bypass surgery. It usually has a surface course and may be easily identified. However, in some cases, the artery passes intramyocardially, or acquires a moderate layer of fat, what makes it difficult for detection.

In a study of seventy patients undergoing coronary artery bypass grafting (CABG), intramyocardial coronary arteries occurred in 17.7%. The most common intramyocardial part (58.6%) represents on the border of proximal and middle portions of the coronary artery [1].

**Figure 1:**
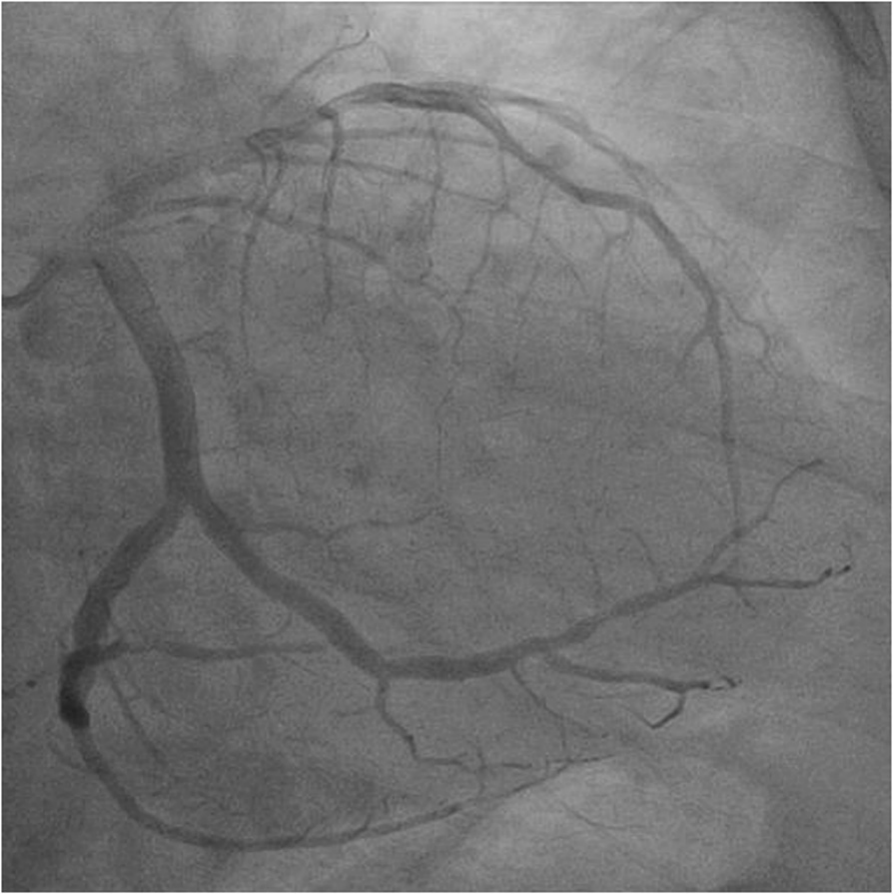
A coronary angiogram showing an intramoycardial left anterior descending artery.

The standard and informative method for cardiac arteriolar system investigation is coronary angiography. Meticulous examination of the angiographic data, especially oblique views, is essential for preoperative identification embedded LAD. The intramyocardial part of the LAD usually dives at an acute angle into the myocardium and appears straight, unlike the serpentine course of the surface vessels (Figure 1) [2,3]. In some cases, a small portion of the artery comes up to the surface, which may be grafted, or may be a reference to locate other place on the artery for further grafting.

Intraoperative investigation of the outer surface of the heart and definition of the main landmarks, arterial and venous branches is the clue to less traumatic determination LAD. It is important to remember that the intramyocardial LAD is usually to the right of the great cardiac vein [2]. Sometimes LAD may be found beneath the small but visible groove on the surface of the heart. That happens because artery embriogenically has underdeveloped connections with the epicardium. When fat accumulates subepicardially, the LAD often pulls the epicardium forming a groove.

In patients with angiographic evidence of an intramyocardial LAD, the course of the vessel should be investigated before aorta cross-clamped [2]. A self-retaining eyelid retractor is a simple, readily available and useful tool for searching embedded vessel.

### Techniques

Many authors have proposed different procedures for determining LAD. Routine dissection approximate location of the artery seems to be the easiest way, but can lead to complications such as penetration into the right ventricle, bleeding from surrounding tissues, formation of compressing hematoma and ultimately failure to find the artery. After gathering available in the literature data, we distinguished five main artery locating techniques that can be used in daily practice (Table 1).

1. G. Robinson [4] introduced a technique involving retrograde passage of a fine atraumatic vascular probe inserted through a distal arteriotomy. The distal part of the LAD, near to the apex, is almost always appears to be superficial. At this place arteriotomy is performed sufficiently to accommodate a 1 mm probe. The probe is gently inserted and advanced proximally until a suitable site for anastomosis formation. It is necessary to keep in mind that sometimes probe can meet obstacles which may represent stenotic, vulnerable segments. By palpation and visual guidance, the artery is opened by longitudinal incision over the intraluminal probe tip. After completion of the anastomosis, the arteriotomy hole is closed with a single gently placed suture or by epicardial tissue imbrication over the arteriotomy, using either interrupted Lembert or Halsted sutures [4-6]. Apostolakis et al. [5] reported use of this technique at off-pump coronary artery bypass grafting (OPCAB), where the probe preferable to keep inside the artery until anastamosis will be completed. This prevents excessive bleeding from the distal arteriotomic hole.

2. I. Gandjbakhch et al. [7] first described the technique of retrograde dissection overlying LAD tissues. R. Parachuri et al. [8], used this technique at 176 patients, and provided more detailed description. The LAD is identified near the apex and exposed proximally by incising the muscle and fat together with Pott’s scissors. The lower scissor blade should be kept exactly on the anterior surface of the artery to minimize the risk of diagonal arteries injure or entering into the right ventricular cavity. The exposure continues until a suitable grafting site is reached. When the apical part of LAD is not visualized artery can be located by tracing the first diagonal artery in the same manner. This technique could be completed by marsupalization of the epicardial edges through whole thickness (epicardium, fat and muscle) with a running 5/0 or 6/0 Prolene suture for better LAD exposure and bleeding control [8].

3. L. Hiratzka et al. [9] in 1986 first described implementation of ultrasound (US) technologies for detection intramurally located coronary artery. It was era of predominantly on-pump CABG on plegic heart, when they proposed to infiltrate tissues around the suspected localization of the coronary artery with cold saline or cardioplegia infusion before scanning. Currently that is no more in a use, as it potentially carries risk of blind vessels injure during infiltration, with subsequent compressing hematoma formation. The use of US Doppler revived with popularity of OPCAB. It has been done number of publications about using probes with different frequencies and other modifications. S. Miwa et al. [10] reported about the better locative capabilities of linear transducers (15-MHz) for the detection of intramural LAD. A. Olearchuk et al. [11] used an epicardial Doppler US (8-MHz) for LAD detection during OPCAB in a patient with thickened neoplastic pericarditis. Oda et al. [12] reported about effective use of color Doppler microprobe for localizing LAD during minimal invasive direct coronary artery bypass grafting (MIDCAB). V. Yuksel et al. [13] noted excellent results for location LAD with pure Doppler probes for easy LAD location by only sweeping on the epicardium. However the US Doppler has its own limitations, mainly due to no-flow conditions, such as arrested heart and proximal LAD occlusion or tight stenosis. Neverthelress, use of Doppler may help assess the flow not only in native arteries, but in grafts and through anastomoses [14-17]. Noninvasive incidental detection intracavitary or very deeply embedded LAD may change the strategy of operation towards conversion from OPCAB to on-pump CABG, to prevent major complications. At cases with planned on-pump CABG and angiographic evidence of embedded LAD, US Doppler may be used before starting cardiopulmonary bypass to give information about approximate vessel location and its condition [14]. Overall, the advancements in medical ultrasonography promise to replace invasive methods artery location with Doppler in the near future [16].

4. U. Aydin et al. [18] reported about locating LAD in two patients using intraoperative cineangiography. The authors used a radiopaque marker (radiopaque tape) for epicardial mapping the anterior surface of the heart. If the marker is not available we advise to use any other tape clipped with ligaclips. The radiopaque agent is infused through the antegrade cardioplegia cannula and a cineangiographic image is obtained from a perpendicular angle using a portable C-arm cineangiographic system. The limitations include requirement an operating room with a set of advanced imaging equipment and use only in patients who are on cardiopulmonary bypass with cross-clamped aorta. Also, it should be used with caution in patients with kidney failure or contrast intolerance. Theoretically, at the hybrid operation room LAD can be detected without using the contrast or cardiopulmonary bypass. The apical part of LAD may be punctured with a needle, through which steel guider can be inserted (same as in the kit for central venous line setting). Radiopaque steel guider along with radiopaque tape would refer for LAD location.

5. In the mid-nineties, it has been described the original way of LAD exposure, which has to be acknowledged. A blunt-ended needle with mounted elastic tape enters deeply into the myocardium, passes under the coronary vessels and comes out on the opposite side. The tape is placed under tension, elevating the intramyocardial vessels, which makes it easier to directly dissect the myocardium over the LAD [2]. This technique carries risk of injuring LAD and its branches and penetration into the right ventricle cavity. Also this type of suture is not widely available and needs to be specially ordered. We believe that this technique can be used in rare cases by highly qualified surgeons or surgeons who are familiar with the latter [19].

**Table 1:** Comparison of different methods for the localization of the LAD

**N**	**Author**	**No. of patients/ complications**	**Technique**	**Advantages**	**Disadvantages**
1	G. Robinson [4]	2/0	Using the probe	Easy and quick	- Intimal damage.
					- Requires closure of the distal arteriotomy, which can produce stenosis.
	R.L. Fisk [6]	18/0			
	E. Apostolakis [5]	26/0			- Identification of the probe.
					- Within the septal.
					- Myocardium can be difficult.
2	I. Gandjbakhch [7]	-/-	Apical dissection LAD in proximal direction	Does not require any special devices	- Penetration of right ventricular chamber
	R.V. Parachuri [8]	176/4			- Injure diagonal arteries.
3	L.F. Hiratzka [9]	2/0	Doppler	Noninvasive	- Need for US with probe.
				Safe	- Not for use on arrested heart.
	K. Oda [12]	5/0			Limited use in proximally occluded or severely stenotic arteries.
	S. Miwa [10]	6/0			
	A.S. Olearchyk [11]	1/0			
4	U. Aydin [18]	2/0	Cineangiography	Simple technique	- Need for hybrid operating theater.
					- Limited use in patients with kidney failure.
					- Only in on-pump patients (with cross clamping).
5	M. Oz	-	Elevating LAD with beneath located elastic tape.	Simple technique	- Risk of injuring the LAD.
					- Risk of penetration into the right ventricle.
					- Need for the special suture.

### Comments and recommendations

The embedded LAD may be an accidental finding on the operating table, the management of which requires special knowledge and experience. Up to now, many various techniques have been proposed for the LAD location. Often the choice of a technique depends on the familiarity and previous experience of the surgeon. Each of these techniques has its own advantages and disadvantages.

Here we developed a stepwise algorithm that can be changed depending on the anatomical features and equipment availability. Basically, it represents stages from the safest to the more invasive.

a. Weighted deliberate examination of the surface of the heart can sometimes be sufficient to detect the artery.

b. If that is not enough, then it is preferable to start from atraumatic methods such as Doppler US or cineangiography, depending on the use of cardiopulmonary bypass.

c. Not all surgical theatres are equipped with advanced medical imaging technologies; also, these methods are not applicable for all cases. Then it is preferably to perform retrograde dissection of the epicard, fat and muscles overlying LAD. The dissection must be strictly vertical to the artery.

d. When the depth of the wound reaches about 4 mm, we recommend stopping dissection, to prevent right ventricle perforation; and to change strategy towards exposure LAD over the fine probe (described above). The use of the probe could be the last resort, as unlike to other techniques, it may adversely affect the vessel intima, what may further lead to early stenosis or arterial thrombosis.

**Figure 2:**
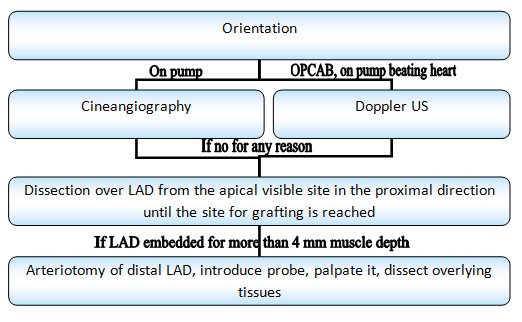
Clinical algorithm for locating the left anterior descending artery during coronary artery bypass grafting.

Figure 2 is showing the clinical algorithm for locating the LAD during CABG.

## Conclusion

Embedded left anterior ascending artery is not uncommon, and intraoperative detection remains a challenge. The technique for LAD exposure should be reproducible, widely available, non- or miniinvasive, implementable for all types of vessel lesions and at the arrested heart. In this respect epicardial ultrasound scanning promising replace other techniques. We propose an algorithm that can serve as a guide, but does not call for absolute obedience, as every case still requires an individual approach.

## Competing interests

The authors declare that they have no competing interests.

## Authors’ contributions

EZ, as the primary author, helped draft the main manuscript; he performed writing, editing, organization, formatting, the paper. He also brought the figure used. HA performed mainly the research and literature review. He reviewed the manuscript and answered the reviewers’ comments. Both authors read and approved the final manuscript.
